# Intersectoral collaboration at a decentralized level: information flows in child welfare and healthcare networks

**DOI:** 10.1186/s12913-022-07810-z

**Published:** 2022-04-06

**Authors:** Mariëlle Blanken, Jolanda Mathijssen, Chijs van Nieuwenhuizen, Jörg Raab, Hans van Oers

**Affiliations:** 1 Tilburg University, TRANZO - Scientific center for care and wellbeing, PO BOX 90153, 5000 LE Tilburg, the Netherlands; 2 Tilburg University, Department of Organization Studies, School of Social and Behavioral Sciences, PO BOX 90153, 5000 LE Tilburg, the Netherlands

**Keywords:** Information exchange, Structures, Integrated care, Child welfare and healthcare networks, Network stability, Density, Degree centralization, Longitudinal multiple case studies, Compare density procedure, QAP correlation procedure

## Abstract

**Background:**

As needs of families with social and behavioral health problems often exceed the expertise and possibilities of a single professional, service or organization, cross-service collaboration is indispensable to adequately meeting those needs. Despite the progressive focus on organizing integrated care, service fragmentation and service duplication remain persistent problems in child welfare and healthcare service delivery systems. A crucial factor to overcome these problems is information exchange between organizations. This study explores and compares the development over time of structures of information exchange in networks, concerning both material and knowledge-based information.

**Methods:**

A comparative case study and social network analysis of three inter-organizational networks of child welfare and healthcare services in different-sized municipalities in the Netherlands. The research population consisted of organizations from various sectors participating in the networks. Data were collected at two moments in time with a mixed method: semi-structured interviews with network managers and an online questionnaire for all network members. Density and degree centralization were used to examine the information exchange structures. Ucinet was used to analyze the data, with use of the statistical tests: Compare Density Procedure and Quadratic Assignment Procedure.

**Results:**

This study shows that different structures of information exchange can be distinguished, concerning both material and knowledge-based information. The overall connectedness of the studied structures of the networks are quite similar, but the way in which the involvement is structured turns out to be different between the networks. Over time, the overall connectedness of those structures appears to be stable, but the internal dynamics reveals a major change in relationships between organizations in the networks.

**Conclusions:**

Our study yields empirical evidence for the existence of and the differences between structures and dynamics of both material and knowledge-based information exchange relationships. With a loss of more than a half of the relations in a year, the relationships between the organizations in the network are not very stable over time. The contrast between major internal dynamics and the stable overall connectedness is an important point of concern for network managers and public officials, since this impermanence of relations means that long-term integrated care cannot be guaranteed.

## Background

As needs of families with social and behavioral health problems often exceed the expertise and possibilities of a single professional, service or organization, cross-service collaboration is indispensable to adequately meeting those needs [1–3]. It is for that reason important that organizations within the child welfare and healthcare service system collaborate sufficiently, for instance by sharing resources such as staff, equipment, information about clients’ conditions and effective treatment. Otherwise, the risk that these families receive an inadequate treatment or fall through the organizational cracks of that system is considerable [4, 5]. To this end, there has been - in the past 10 years - a progressive focus on organizing integrated care through collaborating in cross-sectoral service delivery networks [6–11]. Unfortunately, service fragmentation and service duplication remain persistent problems in child welfare and healthcare service delivery systems [4, 5].

A crucial means to overcome these problems is information exchange between the organizations that constitute a network of welfare and healthcare services [2, 5, 9, 12–16]. There is strong evidence that sharing information - including case reports and substantive expertise - in an accessible and comprehensible way is an important facilitator to provide integrated care [5, 8, 17]. Information exchange between organizations is vital for a shared understanding of families’ needs, a timely response and inter-professional collaboration within a welfare and healthcare service system [2, 17]. Therefore, to get a grip on these key processes and to ultimately achieve an effectively operating care network, insight into the flow of information is essential not only for public management scholars building theory on networks, but also for network managers and public policy officials [4, 18]. One way of achieving a better understanding of information flows is by analyzing the structure of information exchange relationships [19]. In knowledge networks literature, the focus is frequently on the structural properties of networks [20].

Within a network, different structures can exist. Resource dependency theory argues that organizations in a network will interact with those other network members that control access to the resources they need [21]. The type of resource being considered in the interactions affects the structural properties of networks, because it influences the intrinsic characteristics of organizations. These intrinsic characteristics, such as resource dependency and remit of activity, determine the position and role played by organizations in the interorganizational network [22]. As a result, structures of network relationships can be explained by the tangibility of resources being exchanged in the network [23–25]. Examples of resources are staff, equipment, influence, reputation, referrals, and information. The more tangible the resources that are exchanged in a network, the more likely it is that the structure of relationships based on that resource will be centralized around one or a small number of key organizations, as this organization controls (or these organizations control) access to these resources [25]. The exchange of intangible resources, on the other hand, tends to be diffused among several organizations in the network [25]. This distinction in terms of tangibility also applies for the nature of information, ranging from tangible, material informatio*n* (contracts, directives, commissions, and invoices) to intangible, knowledge-based information (verbal case reports, interprofessional consultation regarding clients’ conditions and effective treatment), more referred to as the tacit–explicit dimension of knowledge [20]. Therefore, we expect that there will be different structures of information exchange within a network. However, given the limited prior research on this topic [26], it is unclear whether such structures of information exchange exist within networks and if so, to what extent networks differ amongst each other in this respect.

In addition, networks are not static but dynamic systems [27–29]. Consequently, it is to be expected that information exchange relationships are continuously evolving, as information exchange is one of the key processes in a network [30, 31]. As a network system matures over time, relationships may become more cemented and robust [32, 33]. Such stability of network relationships turns out to be a major factor in explaining network effectiveness regarding client services [34]. Conversely, flexibility is important for ensuring rapid network responses in ways that meet changing families’ needs [35]. However, studies applying longitudinal network analyses in the field of (child) welfare and healthcare services are scarce [4, 26, 36, 37]. Therefore, it is unclear how structures of a network vary over time and whether the relations between the individual organizations, i.e., the internal network dynamics, remain the same over time.

Hence, this study explores and compares the development of structures of information exchange in networks over time, concerning both material and knowledge-based information. The research questions are: 1) To what extent can structures of respectively material and knowledge-based information exchange be distinguished in child welfare and healthcare networks? and 2) To what extent do these overall structures change over time and is that pattern similar to the internal network dynamics?

## Methods

### Research setting

The research field of this study was the societal and administrative context of the Dutch child welfare and healthcare service delivery system. Like many other countries, the Netherlands implemented welfare and healthcare state reforms that shifted key responsibilities from the central to local levels of government [38–43]. Since 2015, municipalities are fully responsible for the child welfare and healthcare service delivery system [44].

In this study, we used a comparative case study approach and social network analysis on three inter-organizational networks of child welfare and healthcare services in different-sized municipalities in the Netherlands [45, 46]. Network I was located in a midsize municipality (around 180,000 citizens), Network II was located in a small municipality (around 66,000 citizens), and Network III covered four very small municipalities that collaborate in providing child welfare and healthcare services (with 13,000–20,000 citizens per municipality, i.e., a total of about 60,000 citizens).

#### Research population

The research population consisted of organizations that participated in the child welfare and healthcare service delivery networks, i.e. network members, with the representatives of these network members as the units of observation [19]. The following definition of a network was used: the network of child welfare and healthcare services consists of organizations with whom the local government, according to the network manager, works together to achieve the main network goal of the Child and Youth Act. Employees who act as boundary spanners between the organizations in the network were the respondents [47, 48]. The network managers - the responsible managers of the municipalities’ child and youth support departments - were asked to identify the network members and to categorize them into different sectors, and to select the boundary spanners.

The networks were composed of organizations from various sectors. Table 1 presents the different sectors and provides examples of organizations and professional groups that belong to a sector. Even though they differ in size, the three networks have the same composition. Network I, with 135 and 132 participating organizations in respectively 2018 and 2019, is the largest network compared to Network II with respectively 86 and 67, and Network III with 75 and 73 organizations. All sectors are present in the networks, except for volunteer organizations in Network II, since the network manager did not list them as network members.

**Table 1:** Sectors and examples of organizations and professional groups in the network

Sectors	Examples of organizations and professional groups
1. Center for youth and family	child and youth welfare and healthcare center
2. Municipal government	youth care expert team, youth and family team, school attendance officers, youth/social support/community service/employment/safety/procurement & contracting departments of the municipal government
3. Basic social organization	social work, welfare work, disabled support, youth and family support, library, food bank, refugee council
4. Education	care coordinators primary and secondary education
5. General practitioners	child and family doctors
6. Health and prevention	child and youth health care center, infant welfare center
7. Childcare and nursery	pre-school, child day-care center, nursery, after school-care including homework support
8. Specialized youth care	youth mental health care, child and youth care, (forensic) psychiatry, orthopedagogy, psychology, disabled childcare
9. Protection & social rehabilitation	youth protection, youth probation officers, juvenile social rehabilitation
10. Safety	police officers responsible for juveniles, protection against child maltreatment, safe houses (crime prevention), public prosecution department, family & youth court, juvenile prison, childcare & protection board, community service supervisor
11. Volunteer organization	Village or ward council, social policy advisory council, informal help for family or neighbors, community center, scouting/music/sport/leisure clubs

Since the individual professionals of some network members operated within a limited working area – such as school care coordinators in education organizations, school attendance officers in municipal organizations, general practitioners (family doctors) and organizations for childcare and nursery – we invited more than one boundary spanner from these network members for the survey. For example, in Network I there were a total of thirty family doctors in the municipality. As the working area of one family doctor was limited to a small part of the municipality, we invited them all to participate in this study. Since the organization is the level of data analysis, we aggregated the results for these boundary spanners to the level of their organization or professional group (see data analysis for information on the applied rules).

For Network I, we also used a threshold for the selection of network members from the sector “specialized youth care organizations”. As a relatively large number of these organizations only had a few juveniles in treatment in 1 year and therefore had peripheral positions in the network, we selected only the organizations that had a minimum of six juveniles receiving care in 2017 (94 of 162 organizations) and in 2018 (92 of 172 organizations). This threshold is generally used for privacy reasons. The final selection of specialized care organizations per network together comprised between 82 and 98% of all juveniles residing in that municipality who received specialized care in the years 2017 or 2018. In this way, we were able to strike a balance between a questionnaire that is manageable for the respondents and yields representative information about the specialized youth care organizations. Table 2 displays the number of network members, including the response rates of the online questionnaire.

**Table 2:** Summary of research population and response

	Network I^a^		Network II^a^		Network III^a^	
	2018	2019	2018	2019	2018	2019
Number of invited network members	135	132	86	67	75	73
Number of responding network members	70	77	49	39	51	44
Response percentage network members	52%	58%	57%	58%	68%	60%

^a^ Network I in municipality with around 180,000 citizens, Network II in municipality with around 66,000 citizens, and Network III in four municipalities with a total of about 60,000 citizens

### Data collection

Data of the three networks were collected at two points in time. The first data collection took place in the period of November 2017 to September 2018 and the second between April to September 2019. Both data collections consisted of two steps. First, semi-structured interviews with the network managers were conducted. The aim of the interviews was to identify the boundaries of the network by determining the network members and categorizing them into different sectors, and to select representatives of the network members as potential respondents for the online questionnaire. Second, an online questionnaire was sent out to the representatives of all the network members, to collect data about both material and knowledge-based information exchange relations between the organizations.

### Measures

To measure relationships between the organizations, the respondents were presented a list of all the organizations of the network and were asked to identify the organizations with which their organization had contact at least once a year, including face-to-face contact (meeting, consultation, conference), by telephone or email. Then, to measure the two types of information exchange relationships between the organizations, the respondents were asked to indicate if their organization had contact with the other organizations specifically for sharing material information (practical information such as official directives, contracts, commissions, annual account, and invoices) and/or knowledge-based information (verbal case reports, and interprofessional consultation regarding clients’ conditions and effective treatment).

Density and degree centralization, as two global measures of network structure [19], were used to examine the pattern of interaction in information exchange structures, for both material and knowledge-based information. Network density indicates the overall connectedness among organizations in the network, while degree centralization shows how the involvement is structured [25]. Density is calculated by dividing the total number of ties in a network by the maximum number of ties possible [49]. The higher the score (ranging from 0 to 1), the more connected the network [50]. Degree centralization is the extent to which links are concentrated (or distributed) among the nodes of the network [51]. It refers to the power and control structure of the network [32, 49, 52, 53]. Degree centralization is calculated as the sum of the difference in centrality between the most central node and every other node divided by the sum of the difference between the theoretically most centralized node and every other node [54]. This denominator represents a star network with one node in the middle connected to every other node (while all the other nodes are not connected). Scores range between 0 and 1, with 1 being the highest possible centralization. In a network with a high level of degree centralization, one or more organizations occupy a more central position than others [51].

### Data analysis

To analyze the data and to calculate the density and degree centralization of the networks, we used Excel and Ucinet [54]. In Excel, the relational data (material and knowledge-based information exchange) were converted into adjacency matrices that were then inserted in Ucinet. To reflect relationships reported by each organizational dyad and in that way capturing “any link”, the networks were “symmetrized” [55]. This method examines “unconfirmed” or unidirectional network ties, which are ties where a respondent identifies a link between their own and another organization, but the other organization does not confirm (including non-response) this collaboration (53 pp. 350–351). We applied the following rule to create the adjacency matrices: a relation between two network members was coded as existing if at least one of the (boundary spanners of the) network members indicated this relation. The missing values were entered as a reciprocal relationship per responding organization (i.e., transposing the column in an adjacency matrix with the corresponding missing rows). This method is known as the procedure of labeled reconstruction [56] to manage non-response. Then, in Ucinet, we computed the global network measures (density and degree centralization) per full network per year.

Subsequently, to compare the overall network structures, we conducted the same analyses of density and degree centralization focusing on only the organizations that are members of the networks in both years (respectively 119, 65 and 71 organizations in Network I, II and III). We used this selection, as statistical tests to compare network structures and over time requires networks with the same actors [57]. To examine whether the connectedness of the material and knowledge-based information exchange structures per network significantly differ from each other and whether the connectedness of the structures significantly changed over time, we used Compare Density Procedure in Ucinet. This procedure uses a bootstrap technique (bootstrap paired sample t-test) to compare the densities of two not necessarily independent networks with the same actors [58].

Finally, to examine the internal network dynamics – i.e., whether the relations between the individual organizations in 2019 were the same as those in 2018 – we used the QAP (quadratic assignment procedure) correlation procedure of Ucinet. QAP identifies the extent of the association in situations where there really is not any systematic connection between the two networks [57]. It compares the observed matching rate of the same type of relationship across two data collection periods (having the same nodes), to the average of a large number of trials in which the actors in the network are randomly matched [25]. As the relations are binary, we used the Jaccard Coefficient. Scores range between 0 and 1, with 0 indicating no overlap and 1 complete overlap between the networks [57].

## Results

As Table 3 shows, the material and knowledge-based information exchange structures are clearly distinguishable per full network. In all three networks, the knowledge-based information structure has more than twice as many relations (ties) between organizations as well as a larger overall connectedness (density) compared to the material information structures. In addition, except for Network II in 2019, the exchange of material information takes place in a more centralized structure than the exchange of knowledge-based information, as the degree centralization scores for the material information structures are higher. In 2019, the knowledge-based information structure of Network III had a relatively high density score (.28 the highest score) coupled with a relatively low degree centralization score (.46 the lowest score).

**Table 3:** Comparative statistics for information exchange structures for the **full** networks in each year

Network	Information exchange structures	Number of ties		Density		Degree centralization	
		2018	2019	2018	2019	2018	2019
Network I *N* = 135 (2018), *N* = 132 (2019)	Material	1090	1082	0.06	0.06	0.71	0.86
	Knowledge-based	2340	2910	0.13	0.17	0.51	0.76
Network II *N* = 86 (2018), *N* = 67 (2019)	Material	572	432	0.08	0.10	0.76	0.67
	Knowledge-based	1230	964	0.17	0.22	0.61	0.76
Network III *N* = 75 (2018), *N* = 73 (2019)	Material	562	636	0.10	0.12	0.65	0.55
	Knowledge-based	1426	1464	0.26	0.28	0.64	0.46

To test the significance of the differences between the material and knowledge-based information exchange structures and the significance of the differences over time, we conducted the same analyses focusing on only the organizations that are members of the networks in both years (respectively 119, 65 and 71 organizations in Network I, II and III). Table 4 presents, per network, the results of the compare density procedure of two types of information exchange structures. For all three networks in both years, there is a significant difference between the densities of the material and knowledge-based information exchange structures. Over time, there was no change in density for material information exchange per network. For knowledge-based information exchange, only the density in Network I increased statistically significantly (from .15 to .19).

**Table 4:** Compare density procedure of information exchange structures for organizations that are members of the networks in **both** years

Network	Information exchange structure	Number of ties		Density	
		2018	2019	2018	2019
Network I (N119)	Material	948	996	0.07	0.07
	Knowledge-based	2106	2634	0.15 ^A^	0.19^AB^
Network II (N65)	Material	426	402	0.10	0.10
	Knowledge-based	880	894	0.21 ^A^	0.22 ^A^
Network III (N71)	Material	526	566	0.11	0.11
	Knowledge-based	1348	1298	0.27 ^A^	0.26 ^A^

^A^significant difference in density between material and knowledge-based information exchange structures per network per year *p* < .01 (two-tailed, bootstrap 5000 samples)

^B^significant change in density over time per structure per network *p* < .01 (two-tailed, bootstrap 5000 samples)

Table 5 presents the degree centralization scores for the three networks, focusing on only the organizations that are members of the networks in both years. For material information exchange, in Network II and III, there was just a small change in network degree centralization from 2018 to 2019. In Network I the degree centralization of the material information structure increased from .72 to .86. Once again, for all three knowledge-based information exchange structures, the degree centralization scores changed over time. In Network I, there is a large increase of degree centralization (from .54 to .75). Network II also saw an increase in degree centralization (from .60 to .76), but the knowledge-based information exchange in Network III became more diffused, as the degree centralization score decreased from .62 to .47.

**Table 5:** Degree centralization scores for information exchange structures for organizations that are members of the networks in **both** years

Network	Information exchange structures	Number of ties		Degree centralization	
		2018	2019	2018	2019
Network I (N119)	Material	948	996	0.72	0.86
	Knowledge-based	2106	2634	0.54	0.75
Network II (N65)	Material	426	402	0.68	0.66
	Knowledge-based	880	894	0.60	0.76
Network III (N71)	Material	526	566	0.63	0.56
	Knowledge-based	1348	1298	0.62	0.47

Beside the changes in the overall structures, the internal network dynamics were examined by calculating the overlap between the structures in both years. Table 6 presents the results of the QAP correlation procedure. There are statistically significant correlations between both material and knowledge-based information exchange structures over time. In Network I, 42% of the knowledge-based information exchange relations between organizations within this structure in 2019 were the same as those in 2018. For Network II and Network III, that is respectively 45 and 50% of the relations. For material information exchange, the sizes of the significant correlation are smaller, ranging from 22 to 39% of the relations.

**Table 6:** QAP Jaccard correlation between information exchange structures in 2018 and 2019 for organizations that are members of the networks in **both** years

	Material information exchange	Knowledge-based information exchange
Network I (N119)	0.224**	0.422**
Network II (N65)	0.394**	0.449**
Network III (N71)	0.285**	0.495**

** *p* < .01 (two-tailed, 2500 permutations)

## Discussion

This study shows that in child welfare and healthcare networks, different structures of information exchange can be distinguished, comprising material and knowledge-based information. The overall connectedness (density) of the studied structures of the networks is quite similar, but the way in which the involvement is structured – degree centralization – turns out to differ between the networks. Over time, the overall connectedness of those structures appears to be stable, but the internal dynamics reveals a major change in relationships between organizations in the networks.

Our findings regarding the first research question of this study generally are consistent with results of earlier research on resource tangibility [24, 25]. Based on the global measures of density and degree centralization, the difference in information tangibility distinguishes significant different structures in the networks. The exchange of knowledge-based information (verbal case reports, interprofessional consultation regarding clients’ conditions and effective treatment) clearly takes place in a more connected and less centralized structure than the exchange of material information (contracts, directives, commissions, invoices). The three studied child welfare and healthcare networks generally show the same pattern. Further analysis of the degree centrality scores per organization shows that the structures of relationships based on material information exchange are centralized around one organization, while for knowledge-based information exchange the relations are centralized around a group of five to six key organizations. This means that in the exchange of material information one organization plays a central role, while in the exchange of knowledge-based information five to six central organizations are closely involved. This structural pattern can be explained by the functions of the organizations in the network that are involved in exchanging material or knowledge-based information. Just the gatekeeper or the municipal government’s procurement and contracting department plays a central role in the exchange of material information. Gatekeepers are organizations that are legally authorized to commission child and youth services covered by the Child and Youth Act. By contrast, the exchange of knowledge-based information involves five to six organizations with various tasks (gatekeeper, signaling and providing services). Thus, despite the relatively high degree centralization scores of the knowledge-based information exchange structures in Network I and II in 2019 (resp. 0.75 and 0.76), the exchange of knowledge-based information is diffused among several functions in the network.

According to resource dependency theory, whoever has control over resources has power over those who need these resources [21]. Based on this logic, we expect that the presence of two highly different information exchange structures within a network could potentially have consequences for the governance of the network, as these different structures influence the power and control mechanisms in the network [34, 49]. Network managers should acknowledge that the diffused exchange of knowledge-based information among several organizations in the network indicates high levels of professional autonomy. That requires a different approach than the highly centralized material information exchange, suggesting a high level of administrative control over the organizations in the network [24]. To further explore to what extent the power and control structure may be influenced by different structures in a network, further research should examine which organizations fulfill a key role and linking-pin position within these structures of the network.

Our findings regarding the second research question of this study stand out. Comparing the networks over time, we found that the information exchange relationships within the networks are not very cemented. With a loss of more than a half of the relations, the relationships between the organizations in the network are not very stable over time. The material information exchange relationships changed significantly; in 2019, only 22 to 39% of these relations were the same as in 2018. This is notable, as the number of material information exchange relations per network are relatively low and with a high degree centralization. As a network matures over time, knowledge and information about network members, especially regarding core organizations, will spread and relationships become more cemented [32]. For that reason, it is to be expected that the highly centralized material exchange relations are relatively easy to stabilize. On the other hand, one should consider that any change in an originally low number of relations will already imply a relatively large loss of relations.

Based on the finding that while the overall connectedness of the networks is relatively stable, the relationships between organizations and the way in which these relationships are distributed change considerably over time, we argue that time matters for child welfare and healthcare networks. Apparently, information exchange structures need more than 3 years to regroup after a major shakeup like a decentralization of the child welfare and healthcare system: a period previously indicated as sufficient time for networks to stabilize [59]. The found instability of relations within the network is relevant, as the welfare and healthcare state reforms were precisely meant to strengthen the relations between the different child welfare and healthcare services [60–62]. In addition, it is known from business and industry sectors that loss of relations is an important factor for social networks, as it leads to a loss of social capital and ultimately affects service sustainability [63]. Accordingly, it is very important to understand the loss of information exchange relationships, especially knowledge-based information exchange relations, since stability in such relations is crucial for interprofessional collaboration and integrated care [2]. To examine whether the time required to stabilize is longer for information exchange relationships or whether these relationships are always flexible, further research should be longitudinal with several measuring points in time.

### Limitations of the study

Several methodological comments can be made regarding this study. First, the network boundaries were determined by the respective network managers of the municipalities. All organizations partnered by a local government to achieve the main network goal of the Child and Youth Act were included. However, there could be other organizations that contribute to the network goal but that do not collaborate with the local government but only with other members of the network. Nevertheless, we chose this strict determination since the application of this clear criterion makes it easier to reproduce the results [36]. Second, as whole network data allows for very powerful descriptions and analyses of social structures, we used the whole network approach which yields the maximum of information [57]. This means that the networks were “symmetrized” in order to reflect relationships reported by each organizational dyad and to capture “any link” [55]. However, as this approach examines unconfirmed ties, it may have led to an overestimation of some network ties, especially for the non-response organizations, which need to be interpreted with caution. Fortunately, except for the general practitioners, all the expected core network members responded. That is positive, as most measures have the greatest bias when more central nodes are missing and the least when peripheral nodes are missing [64]. Most of the non-responders were network members at the periphery of the network, such as the municipal government’s department of safety, organizations for childcare and nursery, or organizations for youth protection and social rehabilitation.

## Conclusion

Our study emphasizes that child welfare and healthcare networks can be defined as complex collaborations with very different information flows, as it provides empirical evidence of the existence of and differences between structures and dynamics of both material and knowledge-based information exchange relationships. Due to the scarcity of longitudinal comparative whole network research in the field and despite the limitations, the strength of this study is a deeper understanding of structures within networks. The discovery of the contrast between the major internal dynamics and the stable overall connectedness has implications for network policy and management. It has implications for what to expect of interprofessional collaboration and the delivery of integrated care, which has been one of the main goals of the decentralization [60–62]. An important point of concern for network managers and public officials is that stability of information exchange relationships is not at all a matter of course. Due to this impermanence of relationships, integrated care cannot be guaranteed, and for that reason, management strategies to build and preserve internal stability should be considered [65].

## Data Availability

The data underlying this article cannot be shared publicly to protect the privacy of the participants but are available from the corresponding author on reasonable request.
